# Resting-state functional connectivity alteration in elderly patients with knee osteoarthritis and declined cognition: An observational study

**DOI:** 10.3389/fnagi.2022.1002642

**Published:** 2022-10-21

**Authors:** Guanwen Lin, Fei Lan, Duozhi Wu, Guanglei Cao, Zheng Li, Zhigang Qi, Yang Liu, Shuyi Yang, Jie Lu, Tianlong Wang

**Affiliations:** ^1^Department of Anesthesiology, National Clinical Research Center for Geriatric Disease, Xuanwu Hospital, Capital Medical University, Beijing, China; ^2^Department of Anesthesiology, Hainan General Hospital, Hainan Affiliated Hospital of Hainan Medical University, Haikou, Hainan, China; ^3^Department of Orthopedics, Xuanwu Hospital, Capital Medical University, Beijing, China; ^4^Department of Radiology, Xuanwu Hospital, Capital Medical University, Beijing, China

**Keywords:** elderly patients, knee osteoarthritis, resting-state functional connectivity, cognitive function, graph theory

## Abstract

**Objective:**

This study is designed to investigate the brain function changed regions in elderly patients with knee osteoarthritis (KOA) and to explore the relationship between neuropsychological tests and resting-state functional magnetic resonance imaging (rs-fMRI) network to clarify the possible mechanism underlying cognitive changes in KOA patients.

**Materials and methods:**

Fifty-two patients aged ≥ 65 with KOA and twenty-two healthy-matched controls were recruited in this study. All participants were given rs-fMRI check. We used graph theory analysis to characterize functional connectivity (FC) and topological organization of the brain structural network. The relationship between FC values, topological properties, and the neuropsychological test scores was analyzed.

**Results:**

Compared with the controls, fourteen edges with lower functional connectivity were noted in the KOA group. Local efficiency and small-worldness of KOA patients decreased compared to the healthy controls. No significant alterations of nodal topological properties were found between the two groups. There was a significant positive correlation between the AVLT-H (L) and the internetwork of default mode network (DMN) (left/right orbitofrontal Superior cortex) and limbic/cortical areas (left/right caudate, right amygdala). AVLT-H(L) was positively correlated with small-worldness and local efficiency.

**Conclusion:**

The results indicated that for elderly KOA patients with declined cognition, topological properties, FC between DMN and subcortical limbic network related regions are significantly decreased compared to healthy controls. These alterations demonstrated a significant correlation with the neuropsychological test scores.

## Introduction

Knee osteoarthritis (KOA) is a highly prevalent and disabling joint disease that is more common in elderly patients. Long-term consequences of KOA may result in declined physical activity, deconditioning, impaired sleep, fatigue, depression, and disability (Sharma, 2021). The percentage of elderly patients with KOA receiving surgical treatment is increasing rapidly. The postoperative cognitive disorder is one of the most common postoperative complications in aged patients, including postoperative delirium, delayed neurocognitive recovery, and postoperative neurocognitive dysfunctions. These complications can result in decreased quality of life. Preventing perioperative neurocognitive disorders is a priority for patients and families. The cognitive reserve of elderly KOA patients before surgery was associated with postoperative functional network and neurocognitive function changes (Jones et al., 2016; Huang et al., 2018). Thus, a preoperative objective evaluation of cognitive function remains very important.

Research showed that as a clinical neuroimaging technique, resting-state functional connectivity (FC) might be a biomarker substitution to detect neurodegenerative diseases (Hohenfeld et al., 2018). The brain was shown to function as several neural networks by analyzing the blood-oxygen-level-dependent signal fluctuations (Rubinov and Sporns, 2010; Bassett and Sporns, 2017). FC is the temporal correlation of a neurophysiological index measured in different brain areas (Biswal et al., 1995) and has been widely used in resting-state functional MRI (rs-fMRI) studies. Altered FC was visible during cognitive impairment (Chabran et al., 2020), anesthesia (Liu et al., 2020), and aging (Pistono et al., 2021) by setting one/multi-seed regions and making the FC a significant indicator for brain functional changes.

Recently, graph theory-based network analysis has been applied to explore brain connectivity within whole-brain networks. It was used to analyze the brain function in larger scale network, considering both the contributions of FC and topology structures (e.g., global efficiency, local efficiency, and degree centrality) (Wang et al., 2010), which may offer important new insights into the structure and function of networked brain systems (Sporns, 2018). A more concise and completed brain network can be presented for studying brain function changes in physiological conditions and disease development. In some cognitively impaired diseases, findings from rs-fMRI have shown impaired connectivity of the default mode network (Koch et al., 2015). Some studies that employed theoretical graph measures to assess alteration of brain networks have demonstrated a reduced degree of centrality parameter in patients with neurodegenerative diseases (Guo et al., 2016). The disassociation of cognitive change from functional brain connectivity change postoperatively was observed (Browndyke et al., 2021). However, few studies have demonstrated the relationship between cognitive function disorder and brain functional network alterations in patients with KOA.

Therefore, this study aimed to investigate the regions of brain function changes to reveal the relationship between neuropsychological tests and rs-fMRI network analysis and clarify the possible mechanism underlying cognitive changes in patients with KOA. We hypothesize that brain FC and topology structure changes in elderly patients with KOA can potentially predict cognitive function degeneration.

## Materials and methods

### Study design

This is a cross-sectional study conducted at Xuanwu Hospital, Capital Medical University, from 1 September 2020 to 31 August 2021 and registered at the Chinese Clinical Trial Registry (Identifier: ChiCTR2000036310). All the procedures have been approved by the Institutional Review Board of Xuanwu Hospital, Capital Medical University (approved No. [2019]-112). All study procedures were carried out in accordance with the Declaration of Helsinki. All patients received written informed consent.

### Patients

In this study, fifty-two patients with knee osteoarthritis with indication for joint replacement were recruited between January 2019 and August 2021 through Xuanwu Hospital, Capital Medical University. Meanwhile, we also recruited twenty-two healthy volunteers for healthy control. The inclusion criteria are as follows: (1) age ≥ 65 years old, (2) more than 6 years of school education, and (3) have knee osteoarthritis with indication for joint replacement. Exclusion criteria are as follows: (1) Montreal Cognitive Assessment-Basic (MoCA-B) score < 19, (2) any history of drug abuse, severe cardiac, cerebrovascular disease, and diseases associated with cognitive impairment, (3) history of neurosurgery or head trauma, (4) severe mental disorders, (5) left-handedness, and (6) contraindications for MRI or unwillingness to complete the MRI.

### Neuropsychological assessment

All participants were given a battery of neuropsychological assessments by a trained neuropsychologist blinded to the study design. Montreal Cognitive Assessment-Basic (MoCA-B) (Chen et al., 2016) assesses nine cognitive domains (executive function, language, orientation, calculation, conceptual thinking, memory, visuoperception, attention, and concentration). Auditory verbal learning test-Hua Shan (AVLT-H) (Guo et al., 2007) was adapted from the California Verbal Learning Test, presenting 12 words over five trials, and the scores on immediate recall, short-delay free recall (5 min), and long-delay free recall (20 min). The Hamilton Rating Scale for Depression (HAM-D_17_) assesses depression severity (Hamilton, 1967). The Hamilton Anxiety Scale (HAM-A) was adapted to present the severity of anxiety neurosis (Maier et al., 1988).

### Magnetic resonance imaging acquisition

For all the seventy-four subjects (fifty-two KOA patients and twenty-two healthy controls), rs-fMRI was finished before the neuropsychological assessment. All the data were collected by the clinical 3.0 Tesla MRI machine (Verio; Siemens Medical Solutions, Erlangen, Germany) with a 32-channel head coil. The head coil was fitted with foam padding and headphones to minimize the influence of scanning noise and head motion before starting. All the participants were told to stay relaxed with eyes closed and not to think of anything. Three-dimensional T1-weighted images were acquired using the following parameters: repetition time (TR) = 1,900 ms, echo time (TE) = 2.2 ms, inversion time (TI) = 900 ms, flip angle = 90°, resolution = 256 × 256 matrix, 176 slices with a thickness of 1.0 mm, slice gap = 0, and voxel size = 1 × 1 × 1 mm. For the BOLD sequences, a rapid-gradient echo sequence was generated for each subject with the following parameters: TR = 2,000 ms, TE = 40 ms, field of view = 240 × 240 mm^2^, flip angle = 90°, section thickness = 4 mm, acquisition matrix = 64 × 64, and 28 slices with the slice gap = 0.

### Functional magnetic resonance imaging preprocessing

Data preprocessing was conducted using a Python-based pipeline tool involving AFNI, ANTs, FSL, and custom python code, known as the Configurable Pipeline for Analysis of Connectomes (C-PAC^1^). The whole analysis was simplified and accelerated by NeuroScholarTM platform (^2^ Beijing Intelligent Brain Cloud, Inc.).

Structural data preprocessing was conducted by the following steps, such as image de-obliquing, re-orientation (right-to-left posterior-to-anterior inferior-to-superior, RPI), skull stripping, normalizing the individual stripped brain to a Montreal Neurological Institute 152 stereotactic space (1 mm 3 isotropic) using linear/non-linear registrations; segmenting the brain into gray matter, white matter, and cerebral fluid (CSF); and constraining the tissue segmentation of individual subjects by tissue priors from standard space obtained from the FMRIB Software Library (FSL^3^).

Functional data preprocessing was conducted by the following steps, including removing the first 10 images and doing the slice-time correction. After re-oblique the images, we re-oriented all the images to RPI orientation. When the skull stripping was finished, the global intensity was normalized to 10,000. After all the above, functional images were registered to anatomical space with a linear transformation and then a white-matter boundary-based transformation and the prior white-matter tissue segmentation from FSL. To remove the head motion artifact, ICA-AROMA was used with partial component regression. To obtain a better data-preprocessing quality, nuisance signal regression was applied, including (1) mean values from the signal in the white matter and CSF derived from the prior tissue segmentations transformed from anatomical to functional space, (2) motion parameters (6 head motion parameters, 6 head motion parameters one time point before, and the 12 corresponding squared items), (3) linear trends, and (4) global signal only for one set of strategies.

### Functional connectivity network construction

The whole-brain functional connectivity (FC) network was constructed as matrix consisting of nodes and edges based on the anatomical automatic labeling (AAL) template, which includes 90 brain regions in the cortex and 26 brain regions in the subcortex. The overall 116 brain regions are represented as “node,” while the correlation between each node is represented as “edge.” The correlation is defined as Pearson’s correlation between the regional mean time series of all possible pairs of brain regions (*R*-value). Prior to statistical analysis, all the correlations were transformed to z-score (Fisher’s r-to-z transformation) to improve normality. In this way, a 116 × 116 correlation matrix based on AAL template was constructed for each subject.

### Network analysis

The graph theory was used to analyze the topological and regional properties of brain networks with MATLAB-based software Gretna Toolbox (Wang et al., 2015)^4^ and visualized by BrainNet Viewer toolbox (Xia et al., 2013)^5^.

We evaluated the global properties of brain network (Achard and Bullmore, 2007) by following measures as clustering coefficient (Cp), shortest path length (Lp), small-worldness, global efficiency, and local efficiency for all participants. The global clustering coefficient is defined as the average of the likelihood of a neighbor-to-neighbor connection. Greater value of Cp represented a stronger local interconnection within a network. The global shortest path length is defined as the average of all of the shortest lengths between each pair of nodes in the network. Smaller value of Lp means a faster brain’s ability to transfer information. The global efficiency is represented as the information transfer efficiency of the network. The local efficiency represents a fault tolerance of the network.

A small-worldness network can be defined by high local clustering, characterized by a high clustering coefficient and low minimum path length between any pair of nodes. Gamma, lambda, and sigma were indices of small-worldness. Gamma (γ) = C_*real*_/C_*random*_ > 1 (C represented cluster coefficient), lambda (λ) = L_*real*_/L_*random*_ ∼ 1 (L represented shortest path length), and sigma (σ) = γ/λ > 1 (Watts and Strogatz, 1998). A high value of sigma indicates a high efficiency of information delivery.

In addition, we used nodal clustering coefficient, nodal shortest path length, nodal efficiency, and nodal local efficiency to describe the regional properties of the functional network (Achard and Bullmore, 2007; Hagmann et al., 2008). The clustering coefficient of a node measured interconnectability of its neighbors. The nodal local efficiency indicated the communication efficiency among its first neighbors when the node is removed. The shortest path length and efficiency of a node quantified the efficiency of parallel information transfer of that node in the network.

### Statistical analysis

Clinical characteristics were compared between the patient and healthy groups by two-sample *t*-tests and chi-squared tests according to data type using GraphPad 8.0 (GraphPad Software; San Diego, CA, USA). Group differences in functional connectivity (FC) were calculated using two-sample *t*-tests in GRETNA. To explore group differences in topological properties, we applied a series of sparsity thresholds (from 0.05 to 0.5, interval 0.05) consistent with a previously published study (Watts and Strogatz, 1998). Two sample *t*-tests were performed on the global and nodal characteristics. False discovery rate (FDR, *q* < 0.05) was used to correct the differences in FC and topological properties. Partial correlation analysis was used to examine associations between FC values, topological properties, and the neuropsychological test scores, conducting with age, gender, and education as covariates (Bozzali et al., 2015; Goldstone et al., 2016).

## Results

### Demographics and clinical data

The final sample included fifty-two elderly patients with KOA (forty women and twelve men) and twenty-two healthy controls (thirteen women and nine men). Demographic characteristics are shown in Table 1. No significant differences between both groups in demographic variables (age and gender) were observed. Education level was significantly higher in the HC group’s patients than that in the KOA group (*P* = 0.023). MoCA-B, AVLT-H (S), and AVLT-H (L) scores in the KOA group’s patients showed significantly lower than that in the HC group (*P* < 0.05).

**TABLE 1:** Demographic and neuropsychological test.

	Healthy controls (*n* = 22)	KOA patients (*n* = 52)	Cohen’s *d*	*P*-value
Age (years)	70.63 ± 4.58	70.27 ± 4.32	0.08	0.753^a^
Gender (male/female)	9/13	12/40		0.120^b^
Education (years)	11.55 ± 3.41	9.75 ± 2.87	0.50	0.023^a^
NRS score	NA	6 (4–7)		
Neuropsychological test				
AVLT-H (S)	8.55 ± 3.19	5.33 ± 1.99	1.21	0.000^a^
AVLT-H (L)	11.50 ± 2.28	4.87 ± 1.94	3.13	0.000^a^
MoCA-B	25.22 ± 2.95	22.88 ± 3.73	0.70	0.011^a^
HAM-D_17_	NA	2 (0–4)		
HAM-A	NA	2 (0–4)		

KOA, knee osteoarthritis; MoCA-B, Montreal Cognitive Assessment-Basic; AVLT-H (S), Auditory verbal learning test-Huashan version for short term; AVLT-H (L), Auditory verbal learning test-Huashan version for long term; HAM-D_17_, 17-item version of Hamilton’s Depression Scale; HAM-A, Hamilton’s Anxiety Scale.

Data are expressed as mean ± SD and median ± inter-quartile range.

^a^The *P*-value was obtained by two-sample two-tailed *t*-test.

^b^The *P*-value was obtained by two-tailed Pearson’s chi-square test.

*P* < 0.05 was considered as statistically significant.

### Differences in functional connectivity

**FIGURE 1:**
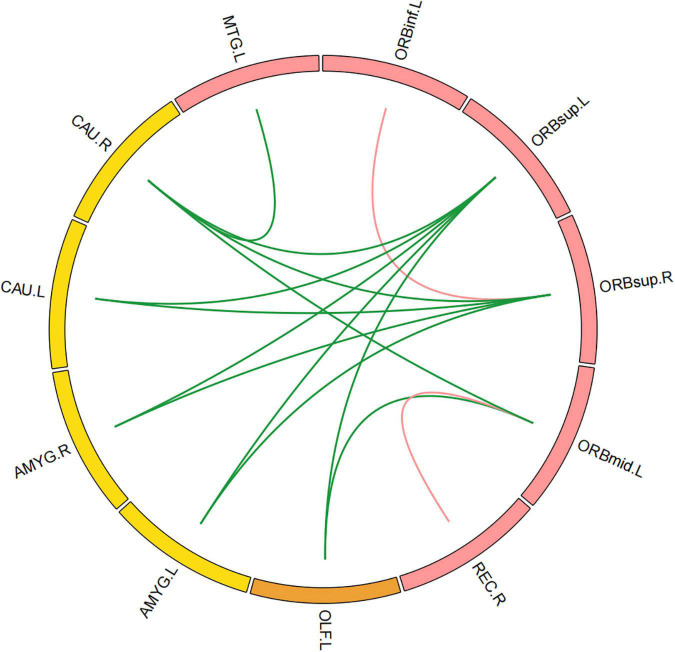
Significant decrease in functional connectivity (FC). The red block represents the default mode network (DMN) regions; the yellow block represents the limbic/subcortical cortex regions; the orange block represents the olfactory cortex. The green line represents the functional connectivity originated from DMN regions; the red line represents the functional connectivity within DMN regions; ORBinf.L, left inferior orbitofrontal cortex; ORBsup.L, left superior orbitofrontal cortex; ORBsup.R, right superior orbitofrontal cortex; ORBmid.L, left middle orbitofrontal cortex; OLF.L, left olfactory cortex; REC.R, right gyrus rectus; MTG.L, left middle temporal cortex; AMYG.L, left amygdala; AMYG.R, right amygdala; CAU.L, left caudate; CAU.R, right caudate.

**FIGURE 2:**
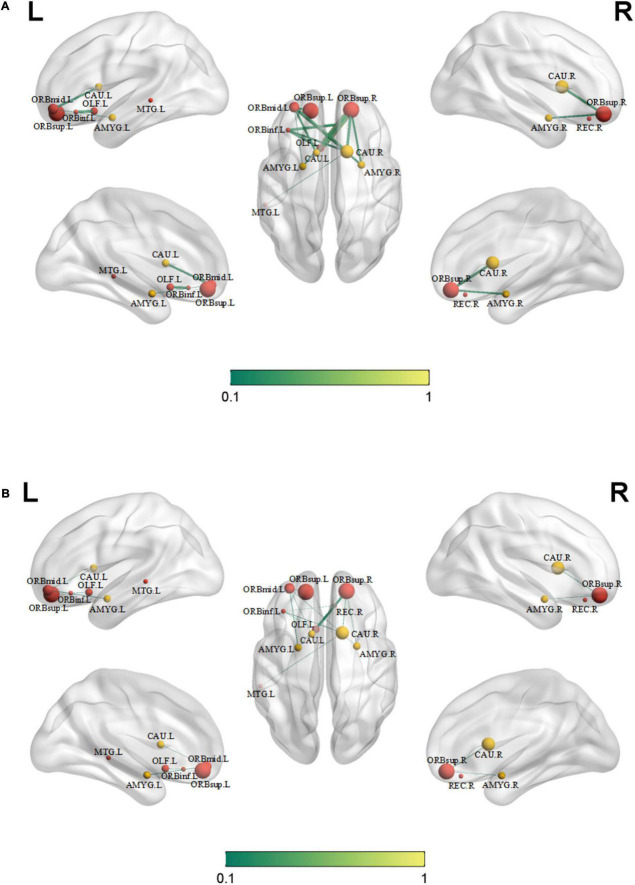
Significant decreased functional connectivity. **(A)** Healthy controls and **(B)** knee osteoarthritis patients. ORBinf.L, left inferior orbitofrontal cortex; ORBsup.L, left superior orbitofrontal cortex; ORBsup.R, right superior orbitofrontal cortex; ORBmid.L, left middle orbitofrontal cortex; OLF.L, left olfactory cortex; REC.R, right gyrus rectus; AMYG.L, left amygdala; AMYG.R, right amygdala; CAU.L, left caudate; CAU.R, right caudate; MTG.L, left middle temporal cortex.

Compared with those in healthy controls, fourteen edges were identified with significantly lower FC in the KOA group’s patients (*p* = 1.12 × 10^–4^, FDR-corrected, Figures 1, 2). Twelve of these edges originating from the default mode network (DMN)-related brain regions (left/right orbitofrontal cortex, left middle temporal cortex) were connected to the limbic/subcortical regions (left/right caudate, left/right amygdala) and olfactory cortex. Two edges were connected within DMN network. Nodes showing significant difference in functional connectivity analysis between the two groups are shown in Table 2.

**TABLE 2:** Nodes showing significant difference in functional connectivity (FC) analysis between HC and KOA groups’ patients (*P* < 0.05, FDR correction).

Nodes	Abbr.	MNI coordinates		
		*x*	*y*	*z*
left inferior orbitofrontal cortex	ORBinf.L	–35.98	30.71	–12.11
left superior orbitofrontal cortex	ORBsup.L	–16.56	47.32	–13.31
right superior orbitofrontal cortex	ORBsup.R	18.49	48.1	–14.02
left middle orbitofrontal cortex	ORBmid.L	–30.65	50.43	–9.62
right gyrus rectus	REC.R	8.35	35.64	–18.04
left middle temporal cortex	MTG.L	–55.52	–33.8	–2.2
left amygdala	AMYG.L	–23.27	–0.67	–17.14
right amygdala	AMYG.R	27.32	0.64	–17.5
left caudate	CAU.L	–11.46	11	9.24
right caudate	CAU.R	14.84	12.07	9.42
left olfactory cortex	OLF.L	–8.06	15.05	–11.46

### Differences in global topological properties

**FIGURE 3:**
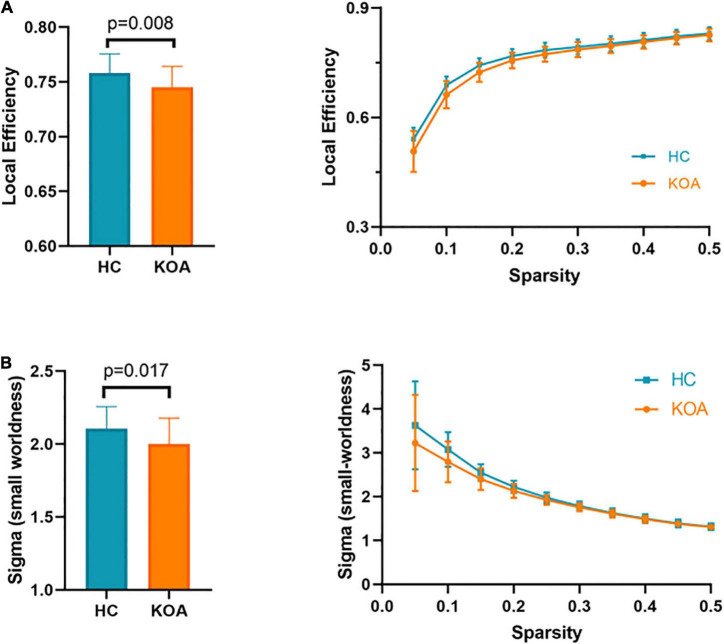
Topological properties. **(A)** Local efficiency. **(B)** Sigma small-worldness.

Patients in both KOA and HC groups were presented small-world organization (σ > 1). Compared with HC group’s patients, small-worldness decreased in the KOA group’s patients (*p* = 0.017, FDR-corrected, Figure 3). Local efficiency in the KOA group’s patients decreased compared with the HC group’s patients (*p* = 0.008, FDR-corrected, Figure 3). No statistical significance was observed in the global efficiency, cluster coefficient, and shortest path length between the two groups.

### Differences in nodal topological properties

The nodal properties including the nodal clustering coefficient, nodal shortest path length, nodal efficiency, and nodal local efficiency were compared. No significant alterations of nodal topological properties were found between the two groups (Supplementary Figure 1).

### Relationships between network characteristics and neuropsychological variables

**FIGURE 4:**
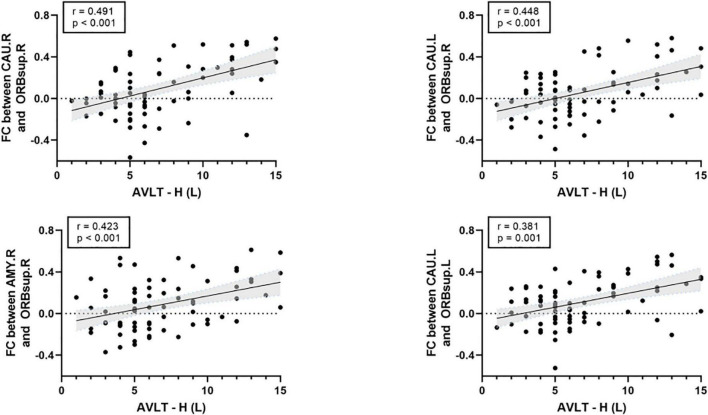
Correlations between cognitive test score and the functional connectivity between default mode network (DMN) and limbic/subcortical cortex. FC, functional connectivity; AVLT-H (L), Auditory verbal learning test-Huashan version for long term; ORBsup.R, right superior orbitofrontal cortex; ORBsup.L, left superior orbitofrontal cortex; CAU.L, left caudate; CAU.R, right caudate; AMYG.R, right amygdala.

**FIGURE 5:**
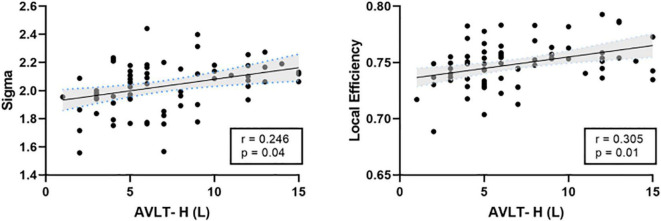
Correlations between cognitive test score and the topological properties. AVLT-H (L), Auditory verbal learning test-Huashan version for long term.

There was a significant positive correlation between the AVLT-H (L) and the internetwork of DMN (left/right orbitofrontal superior cortex) and limbic/cortical areas (left/right caudate, right amygdala) (Figure 4). AVLT-H(L) was positively correlated with small-worldness and local efficiency (Figure 5).

## Discussion

The current study explored the alterations in resting-state FC in patients with KOA by analyzing the rs-fMRI data through graph theory analysis. Our findings indicated that both groups displayed a small-world structure, and global properties of KOA patients, including small-worldness and local efficiency, decreased. Furthermore, these brain network alterations were related to the neuropsychological assessments.

Small-worldness is an important characteristic of a network because it represents an optimal balance between segregation and integration, which is essential for high synchronizability and fast information transmission in a complex network (Watts and Strogatz, 1998). The networks of both HC and KOA groups exhibited characteristic small-world organization, which is consistent with previous studies (Barroso et al., 2021). However, the small-worldness was significantly lower in the KOA group compared to the controls, and this finding indicated the functional brain network of KOA patients is less efficiently organized.

Global efficiency and local efficiency measure a network’s ability to transmit information (Latora and Marchiori, 2001). The local efficiency of the KOA group was significantly lower than that of healthy controls at the whole-brain level, which proved that patients with KOA had abnormal networks with lower local information processing speed, leading to less local efficiency. Interestingly, the two groups found no significant difference in global efficiency. This observation might be due to an over-recruitment of brain regions of mobility declined patients to process complex tasks based on the Related Compensation Utilization of Neural Circuits Hypothesis theory (Reuter-Lorenz and Cappell, 2008). Brain network architecture reorganizes in patients with OA that results from disrupting the whole-brain and local FC by chronic pain (Barroso et al., 2021). Our previous study revealed that end-stage older patients with KOA had decreased amplitude of low-frequency fluctuations (ALFF) in DMN (Lan et al., 2020). The decreased ALFF also proved a local efficiency damage in patients with KOA.

We found the FC network between the DMN [inferior, superior, or middle orbitofrontal cortex (OFC)] and the subcortical limbic network (amygdala, caudate) decreased in older patients with KOA compared to the healthy controls. OFC has a central role in mediating the impact of emotional context on inhibitory control (Buckner and DiNicola, 2019). It is involved in learning stimulus-reward associations (Burke et al., 2008; Zhuang et al., 2021), which receive highly processed sensory information about the current bodily state and emotional and social information (Rudebeck and Rich, 2018). In addition, evidence suggested that the OFC served as a mediator between the prefrontal cortex involved in higher order processing of emotional information and limbic regions involved in emotion perception and generation (Phillips et al., 2003a,b; Beauregard, 2007). Emotional dysregulation and concomitant neurocognitive impairment may be caused by decreased FC between OFC and amygdala of patients with KOA, which was found in our study. Caudate also played a critical role in supporting the planning and execution of strategies and behavior required for achieving complex goals (Grahn et al., 2008). Decreased FC between OFC and caudate may be related to impaired executive function. Patients with KOA suffer from long-term pain, mood disorders, and neurocognitive impairment (Lee et al., 2013). The corticolimbic system, which is involved in emotion and mood processing (Sah, 2017), is also a mediator of chronic pain. KOA pain, as a nociceptive signal, persistently activated corticolimbic circuitry and altered cortex FC due to the structure and function plasticity of the corticolimbic system (McCarberg and Peppin, 2019), resulting in pain chronification.

Functional connectivity also decreased between left olfactory cortex and left superior/middle orbitofrontal cortex. Research showed that olfactory cortex degeneration was correlated with behavioral tests in Alzheimer’s disease and mild cognitive impairment (Vasavada et al., 2015). The entorhinal cortex, which is part of olfactory cortex, has been associated frequently with the DMN (Raichle, 2015). Providing the major input to the hippocampus, entorhinal cortex plays a vital role in memory (Zola-Morgan et al., 1989). These may be a potential reason for memory decline in KOA patients.

The present study used MoCA-B (Luis et al., 2009) to detect cognitive changes and AVLT to detect memory loss (Zhao et al., 2015). Significantly worse scores on neuropsychological tests of MoCA-B and AVLT in the KOA group were observed. These findings suggested that the cognitive function of older patients with advanced KOA decreased compared to healthy controls, consistent with a previous study (Innes and Sambamoorthi, 2018). This study presented that the educational level of the KOA group was significantly lower than the HC group. Evidence that education contributed to cognition changes was controversial. Higher educational level was associated with delayed onset of accelerated cognitive decline (Hall et al., 2007). However, education contributed little to cognitive reserve in old age (Wilson et al., 2019). The exploratory analysis indicated impaired connectivity networks and topological properties significantly correlated with the results of cognitive tests after controlling education as a covariate. FC between the OFC and the limbic or subcortical network positively correlated with AVLT-H (L). Local efficiency and small-worldness were both positively correlated with AVLT-H (L). These results further support the idea that FC and topological structure damages were closely related to memory loss. Altogether, these findings suggested that decreased FC in these important brain area of elderly patients with KOA could contribute to cognitive declination.

The identified positive result in this situation should be reliable with a multiple comparison correction, although the number of patients was limited. The present cross-sectional study cannot address causality. A further longitudinal study is needed to confirm the underlying mechanisms of brain changes in patients and the relationship between cognition and brain network changes in elderly patients with KOA. Future studies combining neuroimaging and specific cognitive domains of older patients with KOA, such as visuospatial abilities, executive functions, and working memory, will be more helpful in exploring their intrinsic association.

## Conclusion

The results indicated that for elderly KOA patients with declined cognition, topological properties, FC between DMN and subcortical limbic network related regions are significantly decreased compared to healthy controls. These alterations correlated with the neuropsychological test scores. Our findings help to improve the understanding of functional network alterations in elderly patients with KOA. Rs-fMRI FC might be employed as a potential biomarker for detecting early cognitive impairment, depression, and anxiety stages. However, this possibility requires further investigation in future studies.

## Data availability statement

The original contributions presented in this study are included in the article/Supplementary material, further inquiries can be directed to the corresponding authors.

## Ethics statement

The studies involving human participants were reviewed and approved by Institutional Review Board of Xuanwu Hospital, Capital Medical University. The patients/participants provided their written informed consent to participate in this study.

## Author contributions

TW, JL, and FL: study design. GL, YL, GC, and ZL: study performance. GL, YL, and ZQ: data analysis. GL and YL: manuscript writing. TW, FL, ZQ, DW, and SY: manuscript revision. All authors contributed to the article and approved the submitted version.

## Footnotes

1
https://fcp-indi.github.com

2
http://www.humanbrain.cn

3
https://fsl.fmrib.ox.ac.uk/fsl/fslwiki

4
https://www.nitrc.org/projects/gretna

5
http://www.nitrc.org/projects/bnv/

## Supplementary material

SUPPLEMENTARY FIGURE 1 Nodal topological properties. No significant alterations of nodal topological properties were found between two groups.
