# Supplementary figures and images for: Resting-state functional connectivity alteration in elderly patients with knee osteoarthritis and declined cognition: An observational study
Source: Front Aging Neurosci. 2022 Oct 21;14:1002642. doi: 10.3389/fnagi.2022.1002642 (PMC9634173; doi:10.3389/fnagi.2022.1002642)

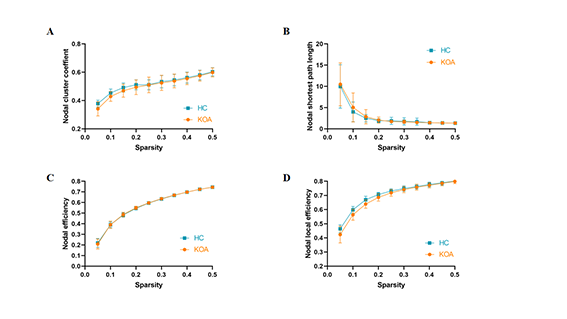

Supplement: SUPPLEMENTARY FIGURE 1 — Nodal topological properties. No significant alterations of nodal topological properties were found between two groups. [file Image_1.TIF]
